# Salinity and hydraulic retention time induce membrane phospholipid acyl chain remodeling in *Halanaerobium congolense* WG10 and mixed cultures from hydraulically fractured shale wells

**DOI:** 10.3389/fmicb.2022.1023575

**Published:** 2022-11-10

**Authors:** Chika Jude Ugwuodo, Fabrizio Colosimo, Jishnu Adhikari, Yuxiang Shen, Appala Raju Badireddy, Paula J. Mouser

**Affiliations:** ^1^Natural Resources and Earth Systems Science, University of New Hampshire, Durham, NH, United States; ^2^Department of Civil and Environmental Engineering, University of New Hampshire, Durham, NH, United States; ^3^New England Biolabs, Ipswich, MA, United States; ^4^Sanborn, Head and Associates, Inc., Concord, NH, United States; ^5^Department of Civil and Environmental Engineering, University of Vermont, Burlington, VT, United States

**Keywords:** membrane adaptation, *Halanaerobium*, fractured shale, lipids, salinity, hydraulic retention time, fatty acid methyl ester

## Abstract

Bacteria remodel their plasma membrane lipidome to maintain key biophysical attributes in response to ecological disturbances. For *Halanaerobium* and other anaerobic halotolerant taxa that persist in hydraulically fractured deep subsurface shale reservoirs, salinity, and hydraulic retention time (HRT) are important perturbants of cell membrane structure, yet their effects remain poorly understood. Membrane-linked activities underlie *in situ* microbial growth kinetics and physiologies which drive biogeochemical reactions in engineered subsurface systems. Hence, we used gas chromatography–mass spectrometry (GC–MS) to investigate the effects of salinity and HRT on the phospholipid fatty acid composition of *H. congolense* WG10 and mixed enrichment cultures from hydraulically fractured shale wells. We also coupled acyl chain remodeling to membrane mechanics by measuring bilayer elasticity using atomic force microscopy (AFM). For these experiments, cultures were grown in a chemostat vessel operated in continuous flow mode under strict anoxia and constant stirring. Our findings show that salinity and HRT induce significant changes in membrane fatty acid chemistry of *H. congolense* WG10 in distinct and complementary ways. Notably, under nonoptimal salt concentrations (7% and 20% NaCl), *H. congolense* WG10 elevates the portion of polyunsaturated fatty acids (PUFAs) in its membrane, and this results in an apparent increase in fluidity (homeoviscous adaptation principle) and thickness. Double bond index (DBI) and mean chain length (MCL) were used as proxies for membrane fluidity and thickness, respectively. These results provide new insight into our understanding of how environmental and engineered factors might disrupt the physical and biogeochemical equilibria of fractured shale by inducing physiologically relevant changes in the membrane fatty acid chemistry of persistent microbial taxa.

**GRAPHICAL ABSTRACT:**
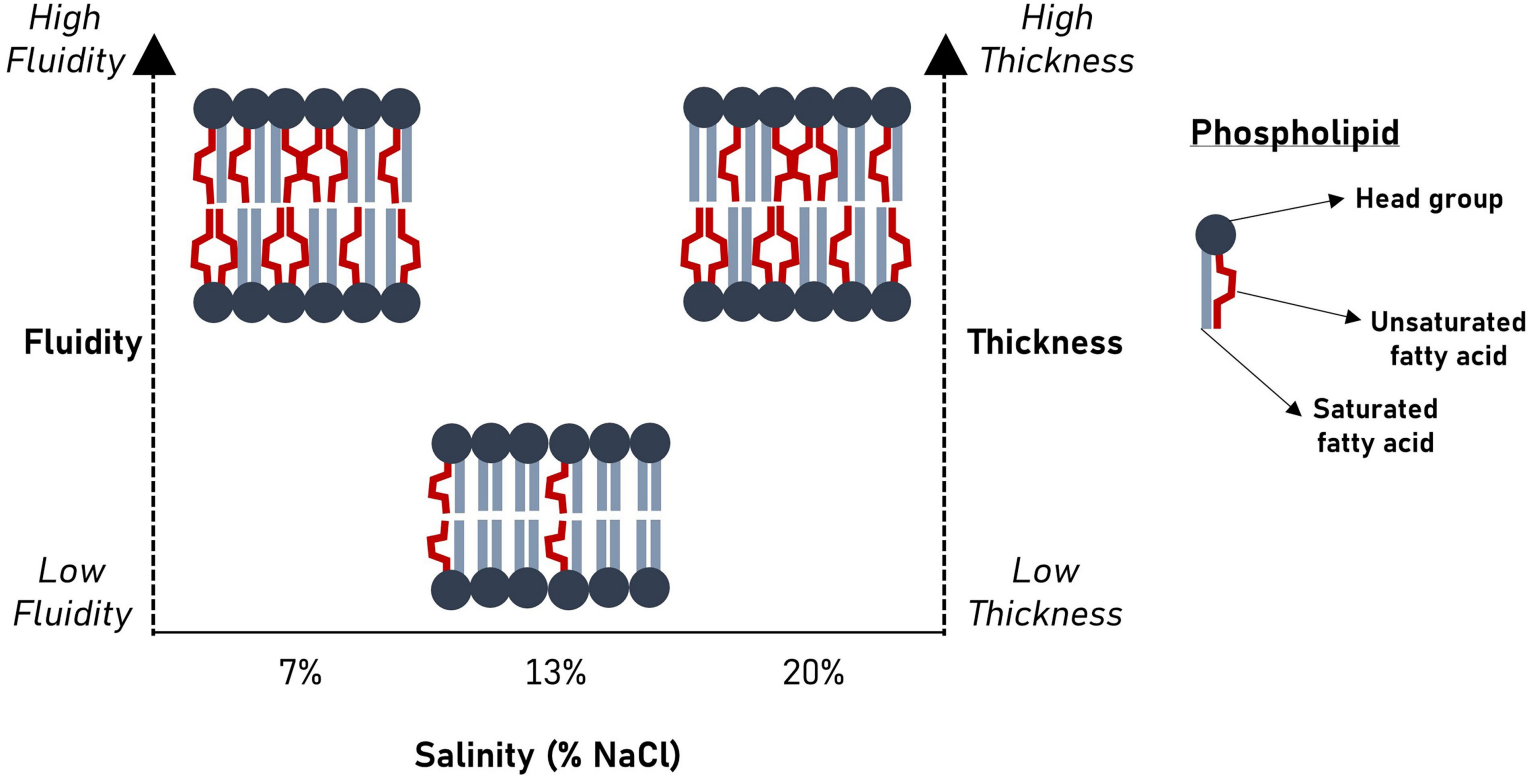
Salinity significantly alters membrane bilayer fluidity and thickness in *Halanaerobium congolense* WG10.

Salinity significantly alters membrane bilayer fluidity and thickness in *Halanaerobium congolense* WG10.

## Introduction

Deep subsurface shale is increasingly being engineered in the United States (US EIA, 2020) and globally using horizontal drilling and hydraulic fracturing, to meet rising demands for energy. Shale reservoirs accounted for 79% of total dry natural gas production in the US in 2020 and are projected to continue to supply most of the dry natural gas through 2050 (US EIA, 2021). During hydraulic fracturing, a water-based “fracking” fluid is injected downhole to extend fracture networks on low-permeability subterranean formations (Stemple et al., 2021). Communities of microbes are introduced into engineered shale with prefracturing fluid, drilling mud, and impoundment water (Gaspar et al., 2014) where they colonize the reservoir (Cluff et al., 2014; Daly et al., 2016). Over time, they become major drivers of subsurface biogeochemistry, with negative consequences for efficient energy recovery and ecosystem health, including biofouling (Booker et al., 2017) and pore clogging (Jones et al., 2021). Fractured shale is a hostile and highly dynamic environment, characterized by a myriad of stressors including brine-level salinities. In addition, well flow rates vary, due to natural deterioration as well as seasonal controls. These unstable environmental and engineered conditions perturb the microbiome, necessitating adaptive changes including adjustments in plasma membrane features. Microbial activities in subsurface energy systems hamper natural gas production, which is a cleaner alternative to other fossil fuels such as coal (Hayhoe et al., 2002; Jaramillo et al., 2007; Burney, 2020). Therefore, to meet the United Nation’s Sustainable Development Goal 7 – affordable and clean energy (Racioppi et al., 2020) – it is imperative to advance our understanding of how persistent taxa in underground hydrocarbon systems respond to ecosystem changes.

Hydraulic fracturing fluids have relatively low salt concentrations, typically <5,000 ppm total dissolved solids (Zeng et al., 2020). However, the salinity of flowback and produced water, which is co-collected with natural gas, ranges from 40,000 to 70,000 mg/L (Zolfaghari et al., 2016), and could be much higher depending on the geochemistry of the formation (Stewart et al., 2015). The high salinity of produced water derives from several geo-physicochemical mechanisms including mixing of fracturing fluids with formation brine (Rowan et al., 2015), and dissolution of salts and minerals on fractured surfaces (Ghanbari et al., 2013; Ghanbari and Dehghanpour, 2015). Members of halotolerant and thermotolerant bacterial and archaeal taxa including *Halanaerobium*, *Marinobacter, Methanohalophilus, Methanolobus*, *Halomonadaceae* and *Halobacteroidaceae*, adapt to these subsurface conditions and dominate the fractured shale ecosystem (Daly et al., 2016). The genus *Halanaerobium* has been identified and recovered from several geographically and geologically distinct subsurface hydrocarbon reservoirs (Jones et al., 2021), indicating it is an important representative taxon in these systems for understanding microbial growth kinetics, roles in subsurface biogeochemistries and responses to physicochemical fluctuations.

Salinity is a topic of global interest (Upadhyay and Chauhan, 2022), transcending engineered subsurface hydrocarbon systems. Notably, salinity affects the productivity of agricultural soils (Singh et al., 2022), thus, threatens food security, which is a critical United Nation’s Sustainable Development Goal (Upadhyay and Chauhan, 2022). Natural causes of soil salinization include mineral weathering, dissolution of fossil salts, rain deposition, and upwards migration of saline groundwater by capillary action (Das et al., 2020). Moreover, anthropogenic management practices especially irrigation, represent a significant source of inorganic salts to soils (Yu et al., 2021). Salinity levels beyond their tolerance thresholds challenge the viability and physiologies of plants and microorganisms, which dominate the biota of the soil matrix. Several studies have linked high salinity to reduced microbial diversity in forest, desert and agro-based systems (Rath et al., 2019; Zhang et al., 2019; Yu et al., 2021). In addition, the availability of micronutrients such as iron (Fe) to plants is impeded by high salt levels (Abbas et al., 2015; Singh et al., 2022). Therefore, salt tolerance is a highly desirable trait in microorganisms and plants in the face of increasing salinization and aridification of global soils. Halotolerant and halophilic species are able to sustain microbial functions and help plants acquire micronutrients whose availabilities are limited in salinity degraded soils (Abbas et al., 2015; Singh et al., 2022; Upadhyay et al., 2022).

The microbial plasma membrane protects the cell from external stressors and mediates critical physiologies, including transport, metabolism, signaling, aggregation and cell-surface interactions (Hurdle et al., 2011). In most microbes, it makes up the cell envelope alongside a peptidoglycan-based cell wall and in a few taxa, other structural layers such as the capsule. In Gram negative bacteria, a second membrane, regarded as the outer membrane (OM) which is rich in lipopolysaccharides, lies outside the thin sheet of peptidoglycan. Most archaea have a single membrane and are covered by a paracrystalline protein layer (Konings et al., 2002; Albers and Meyer, 2011). The plasma membrane is composed of lipids, proteins, and occasionally sugars. A unit membrane is basically a fluid matrix of lipids to which proteins are either attached loosely or enmeshed – the so called “fluid mosaic model” proposed by Singer and Nicolson (1972). The main constituents of the bacterial membrane lipidome are glycerophospholipids which comprise a hydrophilic polar head group covalently linked to hydrophobic fatty acid tails. Phospholipid fatty acids (PLFAs) differ in chain length, saturation, structural configuration, and functional groups. Membrane functions are associated with the activities of peripheral and integral proteins, which in turn depend on biophysical properties such as phase behavior, bilayer symmetry, viscosity, curvature, thickness and elasticity (Chwastek et al., 2020). To a large extent, these properties are collectively dictated by the bilayer lipidome (Klose et al., 2013).

Microorganisms remodel their membrane lipidome to maintain key biophysical properties (Klose et al., 2013; Levental et al., 2017; Chwastek et al., 2020) in response to external stressors. This involves reconfiguration and reorganization of head groups and/or hydrophobic tails. Both fluidity and phase behavior, are important for biological membrane function (Winnikoff et al., 2021). Fluidity affects permeability (Lande et al., 1995) and plays an important role in cellular respiration (Budin et al., 2018), while phase controls lipid raft (floating microdomain) formation (Simons and Vaz, 2004), as well as membrane fusion and budding (Siegel and Epand, 1997). Homeoviscous adaptation, the biochemical mechanism to maintain cell membrane viscosity, mainly depends on the nature of phospholipid fatty acids (Winnikoff et al., 2021). Induced changes in membrane fatty acid composition are common in microorganisms (Fan and Evans, 2015; Chwastek et al., 2020; Winnikoff et al., 2021), including subsurface-dwelling bacteria (Grossi et al., 2010; Fichtel et al., 2015; Roumagnac et al., 2020).

The membrane lipids of moderately and extremely halophilic bacteria are acutely sensitive to salinity (Kates, 1986). However, the effects of salt stress on biological membranes have not been studied as extensively as the effects of temperature and pressure. In halophilic phototrophic bacteria including *Ectothiorhodospira* sp., *Chromatium purpuratum*, *Rhodobacter adriaticus* and *Rhodopseudomonas marina*, salt-induced trends in membrane fatty acid composition were dependent on the optimum growth salinity (Imhoff and Thiemann, 1991). Suboptimal salt concentrations led to acyl chain shortening and increase in unsaturation (Imhoff and Thiemann, 1991). In other halotolerant bacteria, *Vibrio* sp. (Hanna et al., 1984) and *Planococcus* sp. (Miller and Leschine, 2005), proportions of branched chain fatty acids (BCFAs) and cyclic fatty acids increased with salt concentration. These adaptive strategies fluidize the membrane and depress its gel point (Kates, 1986; Winnikoff et al., 2021).

In addition to changes in salinity as the shale well develops, the flow rate of natural gas and produced water is subject to considerable temporal fluctuations. Naturally, constant production leads to an exponential decline in natural gas recovery. In addition, well flow rates are intentionally adjusted according to energy demands from consumers (Sherven et al., 2013). For instance, due to lower demands during warmer months, production of natural gas and co-eluting fluids are typically reduced by “turning back” the well. There is a relationship between well flow rate and fluid residence time in hydraulically fractured shale reservoirs, termed hydraulic retention time (HRT): HRT is increased by lower flows and vice versa. Fluid residence time, in the context of fractured subsurface systems and continuous culture reactors, can affect microbial specific growth rate (Rodrigues et al., 2012) and biomass yields. However, the effects of HRT on biological membranes, especially in high salinity environments, remain largely unexplored.

For *Halanaerobium* and other persistent microbial taxa of fractured shale, salinity, and hydraulic retention time (HRT) are important perturbants of cell membrane structure. Hence, we investigated the effects of salinity and HRT on membrane fatty acid composition and elasticity of *Halanaerobium congolense* WG10 and mixed enrichment cultures from hydraulically fractured wells in West Virginia, United States. This study provides new insight into our growing understanding of how environmental and engineered factors might disrupt the physical and biogeochemical equilibria of fractured shale by inducing physiologically relevant changes in the membrane fatty acid chemistry of persistent microbial taxa.

## Materials and methods

### Growth experiments

Cultures of *Halanaerobium congolense* WG10 (NCBI Assembly accession number: GCA_900102605.1), previously isolated from a Utica-Point Pleasant natural gas well (Booker et al., 2017), were grown in triplicate using chemostat bioreactors (Sartorius Biostat^®^ Q-plus, Germany) at 40°C under three salinities (7%, 13%, and 20%) and three hydraulic retention times (HRTs; 19.2, 24, and 48 h). Produced fluid samples were obtained from the gas-water separator of hydraulically fractured natural gas wells in the Appalachian Basin (Marcellus Shale Energy and Environmental Laboratory – MSEEL, Morgantown, WV). The fluids were filtered on site using 0.45 μm PES filters (EMD Millipore, Burlington, MA, United States) and stored in 1 L sterile amber glass containers. Samples were preserved at 4°C until analysis. Produced fluid enrichment (mixed) cultures were cultivated in triplicate at 40°C under two HRTs (24 h and 48 h). For both culture types: eight (8) days after steady state was attained, cells were pelleted *via* centrifugation at 4,000*g* for 30 min; excess supernatant was removed before storage at −80°C. Tubes containing frozen cells were recovered and left to thaw at room temperature in a laminar flow hood. Then the culture pellets were aseptically transferred to a 15 ml muffled glass tube.

### Lipid extraction and fatty acid methylation

Samples were sequentially extracted ultrasonically according to a modified Bligh and Dyer procedure (Bligh and Dyer, 1959) using three solvent mixtures – dichloromethane (DCM): methanol (MeOH): phosphate buffer, 1:2:0.8 (v/v/v); DCM: MeOH: trichloroacetic acid (TCAA) buffer, 1:2:0.8 (v/v/v); and DCM: MeOH, 5:1 (v/v; Cequier-Sánchez et al., 2008). Phosphate buffer (0.05 M) was prepared by adding 4.35 g of dibasic potassium phosphate (K_2_HPO_4_) with 500 ml of HPLC-grade water and neutralizing to pH 7.4 with 1 N hydrochloric acid. Trichloroacetic acid buffer (0.05 M) was prepared by adding 0.8169 g of TCAA with 100 ml of HPLC-grade water and neutralized with 10 N sodium hydroxide (NaOH) solution to pH of 2.0. Both buffers were washed with DCM (5% of buffer volume) by shaking the mixture vigorously and storing for 5-h at room temperature to allow for complete phase separation.

Exactly 4 ml of DCM: MeOH: phosphate buffer was added to the 15 ml tubes containing culture pellets. To this mixture, 50 μl of 50 pmol per μl of internal standard 1,2-dinonadecanoyl-*sn-*glycero-3-phosphocholine (Avanti Polar Lipids) was added. The tube was shaken, vortexed for 15 s and sonicated in an ultrasonicator bath for 10 min. It was centrifuged for 10 min at 3000 rpm and the supernatant was transferred into a muffled 250 ml glass separatory funnel. This procedure was repeated once with DCM: MeOH: phosphate buffer, and the resulting supernatant was added to the same collecting funnel. The samples were then extracted twice each with DCM: MeOH: TCAA buffer and DCM: MeOH, following the same protocol. The separatory funnel containing the mixture of supernatants was shaken vigorously for 15 s and let to rest overnight to split phase. The organic phase was collected into another muffled separatory funnel, and the aqueous phase was re-extracted with DCM. The pool of organic phases was washed with HPLC-grade water and evaporated to near dryness with a high-purity nitrogen blowdown evaporator at 37°C. The resulting total lipid extract (TLE) was reconstituted with 1 ml of hexane and stored at −20°C until further use.

Total lipid extracts were sequentially fractionated on an activated silicic acid column into fractions of different polarities using hexane, chloroform, acetone, and methanol. The methanol fraction containing phospholipids was evaporated to dryness using a N_2_ gas blowdown evaporator, then resuspended with 500 μl of methanol and 1 ml of methanolic potassium hydroxide. The mixture was vortexed for 30 s and incubated at 60°C for 30 min. After cooling, 2 ml of hexane was added prior to neutralization with 200 μl of 1 N acetic acid. Then, 2 ml of Milli-Q^®^ nanopure distilled water was added to break phase. The samples were vortexed for 30 s and centrifuged for 5 min at 2,000 rpm to separate the phases. The upper (organic) phase was transferred to a muffled volatile organic carbon (VOC) vial and the lower phase was re-extracted with 2 ml of hexane. The solution containing fatty acid methyl ester (FAME) extracts was evaporated to dryness using a N_2_ gas blowdown evaporator and redissolved in 300 μl of hexane. The hexane containing FAMEs was transferred to a GC vial and preserved at −20°C until analysis.

### GC–MS analysis, lipid identification, and quantification

Aliquots (1 μl) of hexane containing FAMEs were analyzed using a Thermo Scientific Trace 1300 gas chromatograph (GC) coupled to a Thermo Scientific ISQ 7000 single quadrupole mass spectrometer (MS). The chromatograph was equipped with a cyanopropylphenyl-based phase column (TRACE™ TR-FAME 30 m, 0.25 mm I.D. × 0.25 μm film thickness), specifically designed for the separation of FAMEs. The GC was programmed to run at 60°C for 2 min, then the temperature was increased at a rate of 10°C per min to 150°C; this was followed by a second ramp to 312°C, at 3°C per min. The final operating temperatures of the injector and detector were 230°C and 300°C, respectively.

FAMEs were identified and quantified using the following external standards (Matreya LLC, State College, Pennsylvania, United States): Bacterial Acid Methyl Ester CP Mixture (BacFAME [1114]), Polyunsaturated FAME Mixture 2 (PUFA-2 [1081]) and Polyunsaturated FAME Mixture 3 (PUFA-3 [1177]). These standards contained FAMEs ranging from 11 to 22 carbons in length and had representative saturates, monounsaturates and polyunsaturates. Identities of FAMEs were initially checked against the NIST17 mass spectral library and confirmed using matching external standards. To quantify FAMEs, each peak was integrated, and its area was compared to the external standard. For FAMEs without matching external standards, the response factor (RF) from the most structurally related FAME standard was used for quantitation (Lewe et al., 2021).

### Calculation of double bond index and mean acyl chain length

Double bond index (DBI) reflects the degree of membrane phospholipid unsaturation and was calculated using the formula (Vornanen et al., 1999):


$$\frac{\sum \left(\mathrm{number of double bonds in fatty acid}\right)\times \left(\mathrm{abundance}\;\left(\mathrm{mol}\%\right)\right)}{\sum \mathrm{abundance}\;\left(\mathrm{mol}\%\right)\;\mathrm{of}\;\mathrm{all}\;\mathrm{fatty acids in the culture sample}}$$

Mean chain length (MCL) was calculated as (Vornanen et al., 1999):


$$\frac{\sum \left(\mathrm{hydrocarbon chain length of fatty acid}\right)\times \left(\mathrm{abundance}\;\left(\mathrm{mol}\%\right)\right)}{\sum \mathrm{abundance}\;\left(\mathrm{mol}\%\right)\;\mathrm{of}\;\mathrm{all}\;\mathrm{fatty acids in the culture sample}}$$

### Atomic force microscopy

To determine membrane elasticity, cultures of *H. congolense* WG10 were fixed onto 0.2 μm polycarbonate membranes (Sterlitech, Kent, WA) by vacuum filtration at 40 psi. Force measurements were performed with MFP-3D-BIOTM Atomic Force Microscope in contact or tapping mode, with polystyrene particle (25 μm) probes with a spring constant of 165.00 pN/nm (Novascan, Boone, IA). A scan rate of 0.15 Hz and a force distance of 1.00 μm were applied. Force map was set at a scan size of 40.00 μm, a scan time of 3.705 min, over a region of four points by four points. Measurements were performed in triplicates, and the pixels related to bacteria were selected empirically based on the range of the elasticity.

### Statistical analyses

Peak intensities were converted to molar concentrations using standard calibration curves, then normalized to percent abundance, sample-wise. All statistical analyses and graphing were done in the R environment version 4.1.2. Normality in data distribution was evaluated using Shapiro–Wilk test. To statistically compare two treatment groups, a two-tailed unpaired Student’s *t*-test was performed. One-way analysis of variance (ANOVA) was applied to comparisons of multiple groups using a Tukey’s honest significance test (HSD) post-hoc analysis. Variations were considered statistically significant at *p* ≤ 0.05.

## Results

### Composition of fatty acid methyl esters

**Figure 1:**
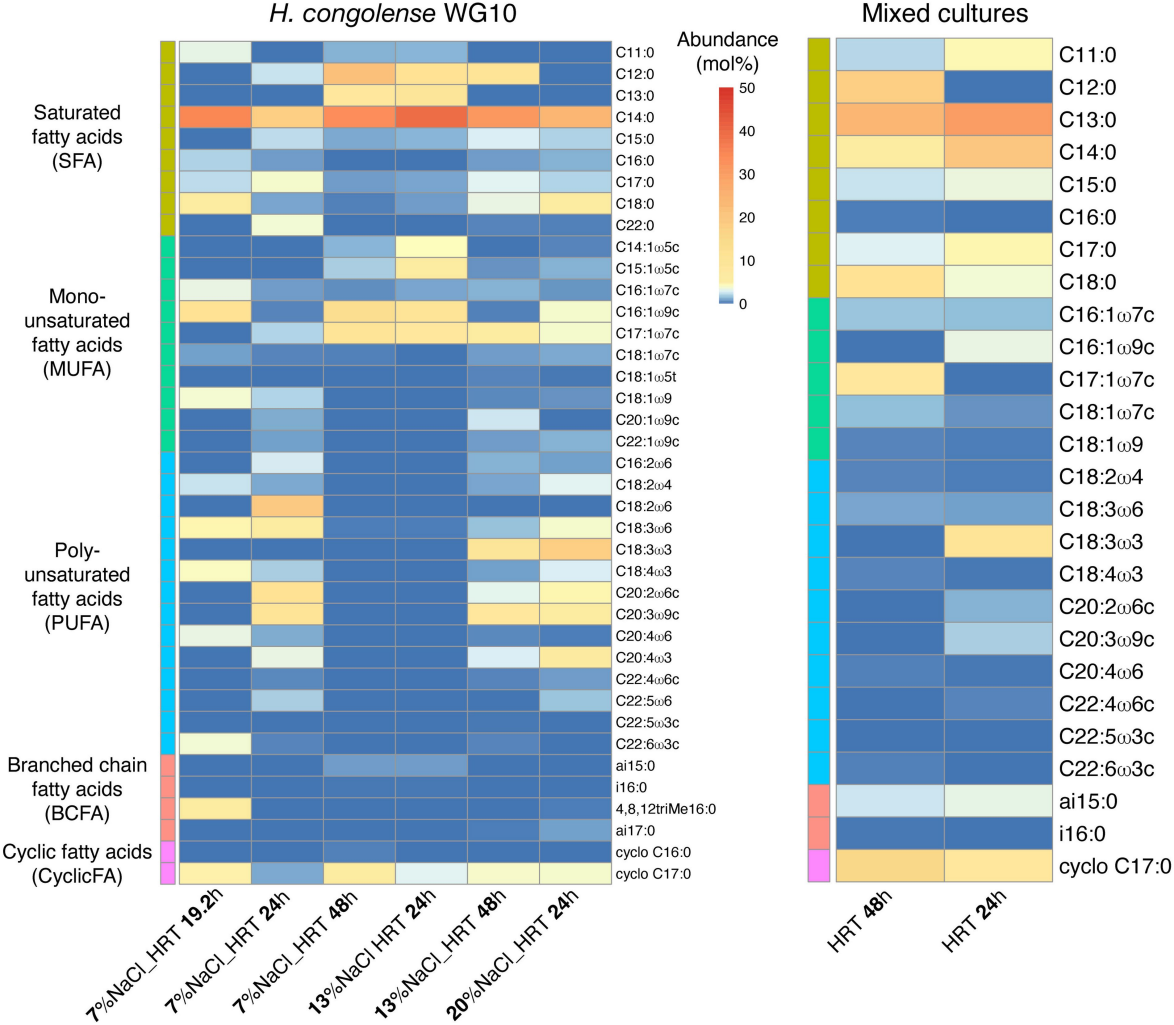
Heatmap distribution of individual phospholipid fatty acids in *Halanaerobium congolense* WG10 and fluid enrichment (mixed) cultures grown at 40°C under different salinities and HRTs. Fatty acids are sorted by class, then chain length and number of double bonds.

We determined relative molar abundances of individual fatty acid methyl esters (FAMEs) that underpin the structural effects of salinity and HRT on the membranes of these dominant shale taxa. Figure 1 shows that in pure cultures of *H. congolense* WG10 cultivated at 40°C under three salinities (7%, 13%, and 20%) and HRTs (19.2, 24, and 48 h), a total of 39 FAMEs were detected. Meanwhile, only 26 FAMEs were found in the produced fluid mixed culture samples enriched at the same temperature (40°C) under similar HRT gradients (24 and 48 h; Supplementary Table).

Among the 39 fatty acids found in *H. congolense* WG10 across treatment conditions, C14:0 had the highest overall mean abundance (~30%). Of the five fatty acid classes identified, saturated fatty acid (SFA) was the most abundant (~51%), followed by polyunsaturated fatty acid (PUFA; ~26%). For the mixed cultures, C13:0 had the highest overall mean abundance (~28%), and similar to *H. congolense* WG10, SFA was the dominant PLFA class (~68%).

**Figure 2:**
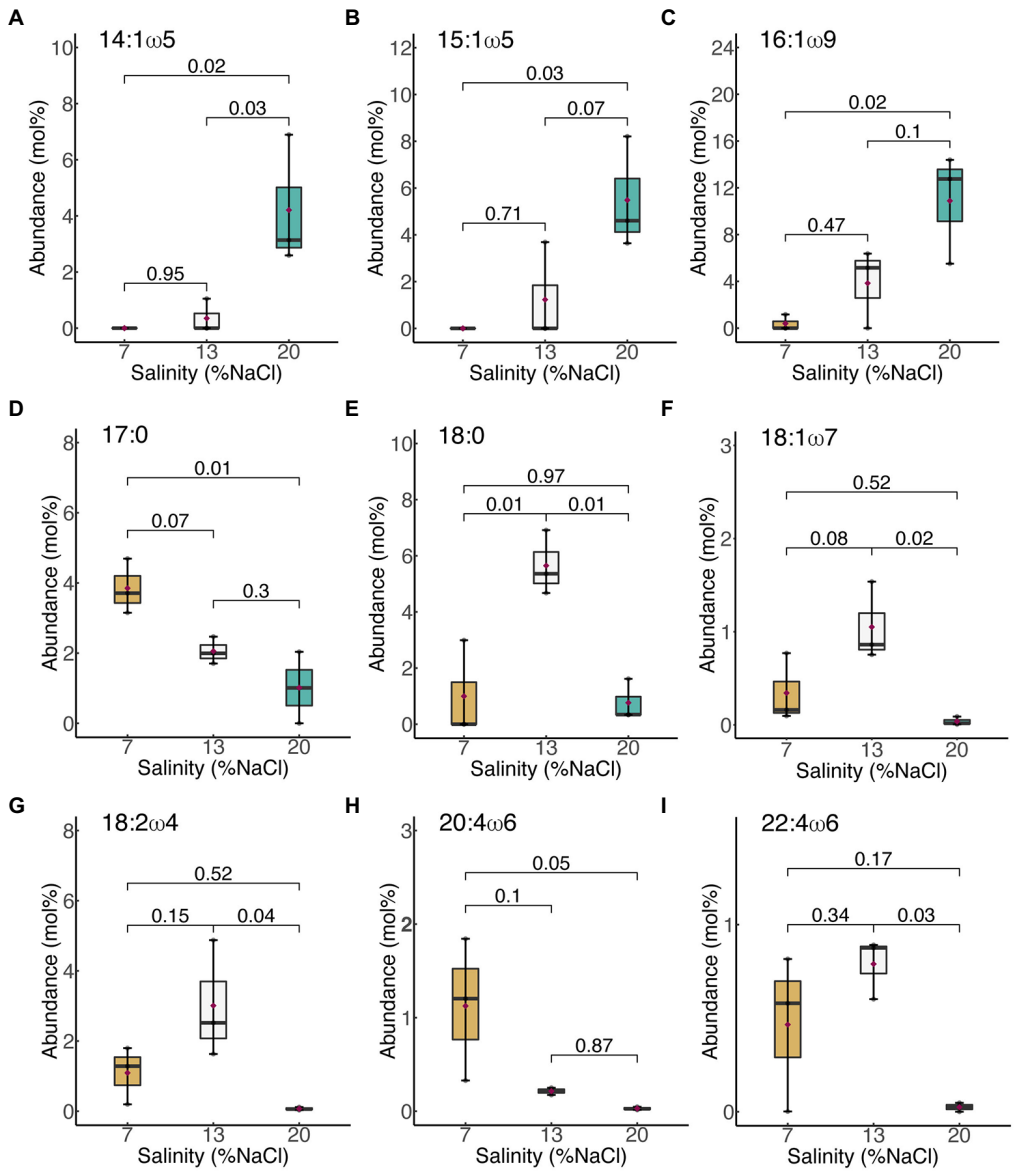
Membrane-derived fatty acids that significantly varied with salinity in *H. congolense* WG10. **(A)** 14:1ω5; **(B)** 15:1ω5c; **(C)** 16:1ω9c; **(D)** 17:0; **(E)** 18:0; **(F)** 18:1ω7c; **(G)** 18:2ω4; **(H)** 20:4ω6; **(I)** 22:4ω6c. Fatty acids are arranged by chain length then by number of double bonds. *p*-Values are obtained from one-way ANOVA of independent groups. Variation is considered significant at *α* ≤ 0.05.

Only nine (9) out of the 39 fatty acids in *H. congolense* WG10 varied significantly with salinity at constant temperature (40°C) and HRT (24 h). Their trends are shown in Figure 2. Among these, three monounsaturated FAMEs [C14:1ω5 (*p* = 0.018), C15:1ω5c (*p* = 0.025) and C16:1ω9c (*p* = 0.023)] significantly increased with salinity (Figures 2A–C). Two others, C17:0 (*p* = 0.011) and C20:4ω6 (*p* = 0.047), significantly progressively decreased as salinity increased (Figures 2D,H). The rest of the fatty acids that varied (C18:0, C18:1ω7c, C18:2ω4 and C22:4ω6c) showed a non-linear pattern of change with salinity – an increase in abundance with increase in salinity from 7% to 13% NaCl, followed by a decline as salinity was further increased to 20% (Figures 2E–G,I). These results imply that unsaturated species are the centerpiece of salinity-induced adjustments to membrane fatty acid chemistry in *H. congolense* WG10. They also indicate complex metabolic exchanges among fatty acids, such as the probable oxidation and desaturation of C17:0 fatty acid to form shorter-chained monounsaturated moieties under increasing salinities.

None of the 39 fatty acids in *H. congolense* WG10 varied significantly with HRT at constant temperature (40°C) and salinity (13% NaCl). Moreover, only one fatty acid, C17:0, showed a significant variation with HRT in *H. congolense* WG10 grown at 40°C and 7% salinity (data not shown). None of the 26 fatty acids found in the mixed cultures varied significantly with HRT (data not shown). This suggests that HRT, which controls cellular growth rate and extent of exposure to toxic metabolic by-products in the reactor, co-ordinately rather than discretely modulates or very minimally influences the plasma membrane fatty acid composition of shale taxa.

### Membrane unsaturation and thickness are increased in *Halanaerobium congolense* WG10 under nonoptimal salinities, but are variably affected by HRT

**Figure 3:**
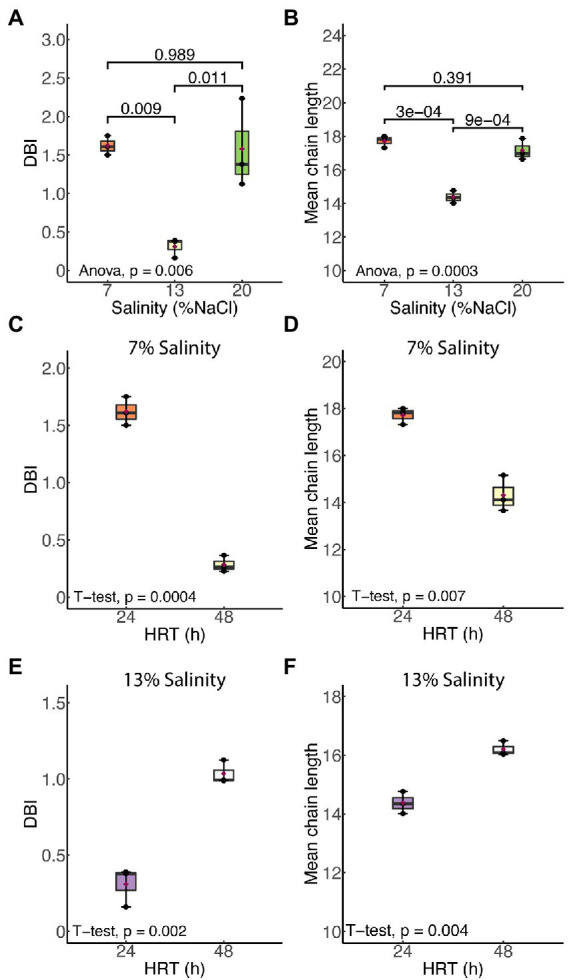
Mean chain length (MCL) and double bond index (DBI) of membrane phospholipids in *H. congolense* WG10 responded distinctly to salinity **(A,B)** and hydraulic retention time (HRT). The effect of HRT was evaluated at two discrete salinities – 7% **(C,D)** and 13% **(E,F)**. *p*-Values are obtained from one-way ANOVA of multiple independent groups or Student’s *t*-test of two independent groups. Variation is considered statistically significant at *α* ≤ 0.05.

Figure 3 shows the effects of salinity and HRT on the mean chain length (MCL) and double bond index (DBI) of membrane phospholipids in *H. congolense* WG10. Both parameters varied significantly with salinity at constant growth temperature (40°C) and HRT (24 h; Figure 3A). As salinity increased from 7% to 13% NaCl, there was a significant decrease in both mean chain length (*p* = 0.0003) and DBI (*p* = 0.009). However, a further increase in salt concentration from 13% to 20% produced the opposite effect, where both mean chain length and DBI increased. There was no significant difference in chain length or DBI between the two nonoptimal salinity conditions (7% and 20%). *Halanaerobium congolense* WG10 also adjusted the mean chain length and DBI of its membrane phospholipids in response to HRT at constant temperature (40°C) and salinity (7% or 13%). At 7%, MCL and DBI significantly decreased with HRT (Figure 3B), while at 13% salinity, *H. congolense* WG10 significantly increased the MCL and DBI of its membrane as HRT increased (Figure 3C). There was no significant variation in either MCL or DBI with HRT (24 versus 48 h) in the mixed cultures (Supplementary Figure S1).

### SFAs and MUFAs in the plasma membrane of *Halanaerobium congolense* WG10 are exchanged for PUFAs under nonoptimal salinities

**Figure 4:**
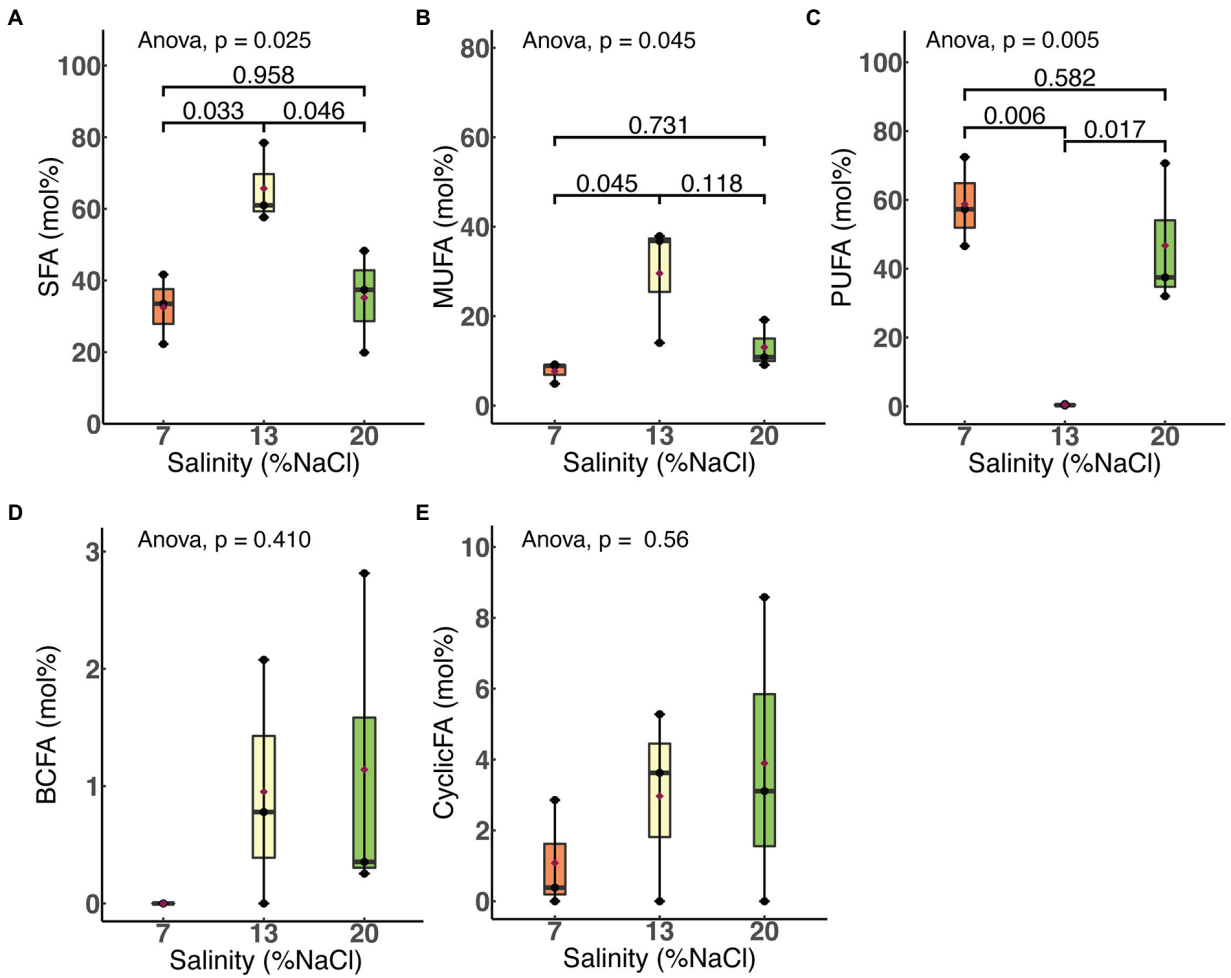
Variations in molar abundances of membrane phospholipid fatty acid classes in *H. congolense* WG10 with salinity at constant temperature (40°C) and HRT (24 h). *p*-Values are obtained from one-way ANOVA of independent groups. Variation is considered significant at *α* ≤ 0.05. SFA, Saturated fatty acid; MUFA, Monounsaturated fatty acid; PUFA, Polyunsaturated fatty acid; BCFA, Branched chain fatty acid; CyclicFA, Cyclic fatty acid.

We examined the effects of salinity and HRT on the relative molar abundances of each of five major fatty acid classes: saturated, monounsaturated, polyunsaturated, branched chain and cyclic. As shown in Figure 4, a significant increase in the membrane saturated fatty acid (SFA; *p* = 0.03) content of *H. congolense* WG10 was observed when salinity was increased from 7% to 13% NaCl. This was accompanied by a more significant decline (*p* = 0.006) in the polyunsaturated fatty acid (PUFA) fraction. With a further increase in salinity from 13% to 20%, SFA significantly dropped (*p* = 0.046) while PUFA increased (*p* = 0.017). The membrane monounsaturated fatty acid (MUFA) composition of *H. congolense* WG10 varied similarly as the SFA fraction, except that its decline as salinity increased from 13% to 20% was not statistically significant. There was no significant variation in all three PLFA classes between 7% and 20% salinities. Changes in the molar abundances of branched chain fatty acids (BCFAs) and cyclic fatty acids, with salinity, were not statistically significant.

**Figure 5:**
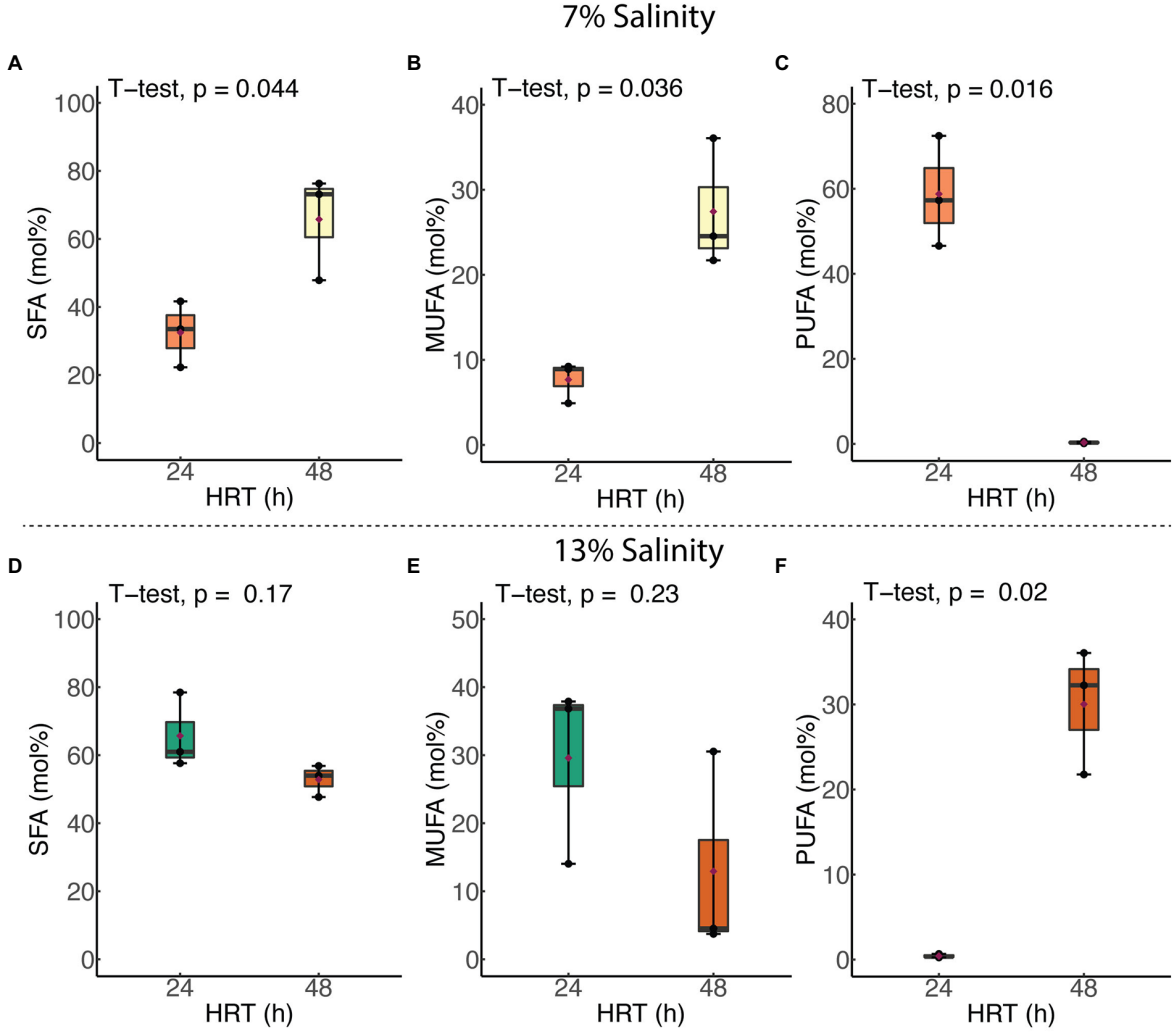
Variations in abundances of membrane PLFA classes in *H. congolense* WG10 with hydraulic retention time at constant temperature (40°C), evaluated at two different salinities – 7% **(A–C)** and 13% **(D–F)**. *p*-Values are obtained from student’s *t*-test of independent groups. Variation is considered significant at *α* ≤ 0.05. SFA, Saturated fatty acid; MUFA, Monounsaturated fatty acid; PUFA, Polyunsaturated fatty acid.

Membrane SFA and MUFA compositions of *H. congolense* WG10 grown at constant temperature (40°C) and salinity (7%), increased significantly as HRT was increased from 24 to 48 h. This was accompanied by a significant decline (*p* = 0.016) in the PUFA content (Figure 5A). Relative molar abundances of both BCFA and cyclic FA did not significantly vary with HRT (data not shown). In contrast, when grown at the same temperature and 13% NaCl, none of the membrane fatty acid classes in *H. congolense* WG10 varied significantly with HRT, except for PUFA whose abundance significantly increased as HRT was increased from 24 to 48 h (Figure 5B). For the mixed cultures, HRT did not produce significant variations in the molar abundances of any of the fatty acid classes (Supplementary Figure S2).

### Membrane elasticity in *Halanaerobium congolense* WG10 does not significantly vary with salinity and HRT

To couple changes in fatty acid composition to a physiologically-relevant aspect of membrane mechanics, we measured the bilayer elasticity of *H. congolense* WG10 grown at 40°C under varying salnities (7%, 13%, and 20% NaCl) and hydraulic retention times (19.2, 24, and 48 h), using atomic force microscopy (data not shown). ANOVA revealed that neither salinity nor HRT induced significant changes in membrane elasticity. However, mean Young’s Modulus (a measure of stiffness) at 13% salinity was considerably lower than at 7% and 20%, a similar pattern of variation with membrane polyunsaturated fatty acid composition, suggesting that PUFA modulates membrane elasitcity in *H. congolense* WG10.

## Discussion

### Salinity and hydraulic retention time influence membrane fatty acid chemistry in *Halanaerobium congolense* WG10

Our findings show that *Halanaerobium congolense* WG10 remodels its membrane fatty acid composition in response to variations in salinity and hydraulic retention time (HRT). To curtail the confounding effect of changes in the microbial lipidome due to growth phase progression (Berezhnoy et al., 2022) rather than induced by bioreactor growth conditions, we used a chemostat system for culture cultivation and harvested cells after they had attained steady state at which point, the specific growth rate is constant and equal to the dilution rate. Being a continuous culture system, cultures were sustained in a prolonged exponential growth phase until harvested at steady state. In general, salinity had a more pronounced and consistent impact than HRT. We used mean chain length (MCL) and double bond index (DBI; Figure 3) as proxies to quantify the effects of both perturbants on critical aspects of cell membrane structure and properties, in lieu of biophysical experimentation. DBI is a measure of degree of unsaturation and by implication bilayer viscosity/fluidity (Berezhnoy et al., 2022). Chain length, on the other hand, estimates membrane thickness. Fluidity and thickness affect the biological functions of the membrane. Salinity and HRT induced significant changes in membrane fatty acid composition of *H. congolense* WG10 which reflected on mean chain length and DBI. Accordingly, the membrane appeared to be thicker and more fluid under low (7% NaCl) and high salt stresses (20% NaCl), compared to optimal salinity (13% NaCl). Also, at optimal salinity, membrane thickness and fluidity increased with HRT. The specific growth rate of cultures in a chemostat is lower at higher HRT. Therefore, our fluidity trend observation contrasts in principle with the findings of a prior study which reported that saturated fatty acid (SFA)/double bond equivalent (DBE) ratio increased as the growth rates of two *E. coli* strains plateaued into the stationary phase (Berezhnoy et al., 2022). This indicates that, in the current study, because growth rate variations were domiciled within the prolonged log phase, membrane lipidome changes is due to other growth-related metabolic distresses rather than stationarity. Taken together, our findings imply that a thicker and fluidized membrane is essential to effective adaptation of *H. congolense* WG10 to osmotic and metabolic stresses. In contrast, membrane fatty acid composition of the mixed cultures of persistent shale taxa was not significantly altered by HRT (Supplementary Figures). It is rational to think that in general, shale microbial communities are better able to resist the impacts of ecological disturbances (external stressors) than isolated species, hence the minimal adaptive changes in membrane chemistry.

Thirty-nine (39) phospholipid fatty acids were found at variable abundances in *H. congolense* WG10 grown at 40°C under different salinities (7%, 13%, and 20%) and hydraulic retention times (19.2, 24, and 48 h; Figure 1). These fatty acids belong to five classes – saturated fatty acid (SFA), monounsaturated fatty acid (MUFA), polyunsaturated fatty acid (PUFA), branched chain fatty acid (BCFA) and cyclic fatty acid (CFA). The relatively low amounts of BCFAs and CFAs could be due to the maintenance of cultures in a prolonged exponential growth phase in the chemostats. Cyclopropanation has been reported to increase with growth progression in *E. coli* such that the lipidome is dominated by lipids with CFA chains during the late stationary phase (Berezhnoy et al., 2022). Only 26 of these 39 fatty acids were found in the mixed culture samples grown at 40°C under varying HRTs (24 and 48 h; Figure 1). We believe that the fatty acid compositional trends observed in these shale taxa are mainly driven by lipid metabolism as opposed to “diet.” Metabolic pathways for fatty acid biosynthesis and post-synthetic modifications exist in bacteria and begin with the conversion of acetyl-CoA to malonyl-CoA (Cronan and Thomas, 2009). After malonyl-CoA condenses with an acyl carrier protein (ACP), the central pathway devolves into several branches that lead to the synthesis of major fatty acids. Fatty acid metabolism in all organisms is globally regulated at various genetic and metabolic levels (Fujita et al., 2007). Faced with changing subsurface conditions, *H. congolense* WG10 and other persistent shale taxa potentially deploy these regulatory mechanisms to modulate fatty acid composition, to maintain functionally-relevant biophysical properties of the plasma membrane.

Membrane polyunsaturated fatty acid (PUFA) composition did not correlate with salinity in *H. congolense* WG10 grown at 40°C and 24 h HRT. Bilayer PUFA abundance during growth at 13% NaCl was significantly lower than at 7% and 20% (Figure 4). Considering that 13% is the optimal growth salinity, this trend indicates that *H. congolense* WG10 adapts to low (7%) and high (20%) salt stress by increasing its membrane PUFA composition. Similarly, a halotolerant bacterium, *Rhodococcus erythropolis*, had significantly higher amounts of membrane derived PUFAs when grown under low (1%) and high (7.5%) osmotic stress, compared to growth at optimal salinity (2.5%; de Carvalho et al., 2014). The observation that none of the individual PUFAs which varied significantly with salinity in *H. congolense* WG10 (C18:2ω4, C20:4ω6, and C22:4ω6c) followed this trend (Figure 2) suggests that they might be functionally interchangeable and regulated as a group rather than as discrete entities (Winnikoff et al., 2021).

Previously thought incapable (Okuyama et al., 2007), bacteria (especially halophiles and psychrophiles) are now well known to incorporate polyunsaturated fatty acids (PUFAs) into their membrane lipidome (Russel and Nichols, 1999; Nichols and McMeekin, 2002; Jadhav et al., 2010; de Carvalho et al., 2014; Moi et al., 2018). Accordingly, we found 14 PUFAs in *H. congolense* WG10 and 10 in the mixed cultures. Their chain lengths ranged from 18 to 22 and number of double bonds varied from 2 to 6. We believe functional metabolic pathways for *de novo* synthesis of PUFAs exist in these persistent shale taxa. Generally, there are two pathways for PUFA synthesis in bacteria – aerobic and anaerobic. The aerobic mechanism begins with a saturated fatty acid and involves repeating steps of desaturation and elongation (Zhu et al., 2006). On the other hand, the anaerobic pathway involves the actions of polyketide synthase (PKS)-related enzymes, also called PUFA synthases (Zhang et al., 2021). A combination of genetic and biochemical approaches is required to confirm the existence and operation of one or both pathways in *H. congolense* WG10. Meanwhile, an NCBI nucleotide search confirmed the presence of a regulator of polyketide synthase expression gene (GenBank: PUU90574.1) in a metagenome-assembled genome (MAG) belonging to *Halanaerobium* sp. isolated from a hydraulically fractured shale well, suggesting this pathway may exist in this genus.

Shale microbes may also possibly acquire exogenous polyunsaturated fatty acids (PUFAs) from their surroundings. This scavenging behavior is not unthinkable among microbes and have been reported in several bacterial species including halotolerant *Vibrio* (Smith et al., 2021), as well as *Pseudomonas*, *Acinetobacter*, *Escherichia and Klebsiella* (Eder et al., 2017; Moravec et al., 2017; Baker et al., 2018; Hobby et al., 2019; Herndon et al., 2020; Zang et al., 2021). In this hypothetical scenario, exogenous PUFAs would likely come from the oil and gas reservoirs. Organisms of the candidate phyla radiation (CPR) and DPANN radiation residing within the deep continental subsurface have been suggested to scavenge, use and modify molecular lipids from external sources (Probst et al., 2020).

The effects of HRT (24 and 48 h) on membrane PUFA composition of *H. congolense* WG10 is discriminated by salinity (Figure 5). When growing at 7% NaCl, total PUFA abundance declines significantly with HRT whereas at 13%, it increases with HRT. Hydraulic retention time (HRT) – the inverse of dilution rate – is a critical microbial growth parameter in continuous culture systems. At abnormally high HRT (in this case 48 h), the dilution rate likely falls below the bacterium’s maximum specific growth rate, upsetting the exponential phase dynamics. Despite being a self-adjusting system, with longer medium residence time in the chemostat, there is substrate depletion and possibly accumulation of toxic metabolic by-products (Foustoukos and Pérez-Rodríguez, 2015). These conditions exert physiological stress on *H. congolense* WG10, prompting membrane acyl chain remodeling to achieve desired biophysical attributes of the bilayer. This translates to increasing membrane PUFA composition when growing at optimal salinity (13% NaCl), but the opposite when subjected to hypoosmotic stress (7% NaCl; Figure 5). We are not exactly sure why this discrepancy exists.

Like PUFA, membrane saturated fatty acid (SFA) and monounsaturated fatty acid (MUFA) compositions of *H. congolense* WG10 did not correlate with salinity at constant temperature (40°C) and HRT (24 h). However, unlike PUFA, molar abundances of SFA and MUFA at 13% NaCl were significantly higher than at 7% and 20% (Figure 4). This inverse relationship between PUFA and SFA/MUFA implies that when confronting low (7%) or high (20%) salt stress, *H. congolense* WG10 exchanges significant amounts of SFAs and MUFAs in its membrane lipidome with PUFAs. This exact same response was reported in the halotolerant bacteria, *Rhodococcus erythropolis* (de Carvalho et al., 2014). Similarly, in *H. congolense* WG10 growing at optimal salinity (13%), membrane PUFA composition was significantly increased under high HRT, even though concomitant reductions in the abundances of SFAs and MUFAs were not statistically significant (Figure 5B).

It is our hypothesis that, just like other bacteria (de Carvalho et al., 2014), *H. congolense* WG10 constitutively expresses desaturases and elongases, which are quickly activated when needed to convert saturated and monounsaturated fatty acids to polyunsaturated fatty acids. We believe that stearoyl-CoA desaturase plays a key role in membrane PUFA biosynthesis in *H. congolense* WG10 growing under salt-stressed conditions, based on the observation that out of the 7 SFAs and MUFAs that varied significantly with salinity (Figure 2), only C18 fatty acids – C18:0 and C18:1ω7c – were downregulated at 7% and 20% salinity compared to 13%. (Figure 2). Stearoyl-CoA desaturase introduces double bonds into C18 fatty acyl chains. Many marine bacteria express elongases and desaturases, including the soluble stearoyl-CoA desaturase and membrane-bound acyl-CoA desaturases (Moi et al., 2018; Berezhnoy et al., 2022).

### Rationalizing membrane acyl chain remodeling in *Halanaerobium congolense* WG10

Now, we turn to common hypotheses of membrane lipidome remodeling to attempt a rationalization of the responses of *H. congolense* WG10 to changes in salinity and hydraulic retention time (HRT). Both factors are relevant for hydraulic fracturing of deep subsurface shale and appeared to exert selective forces on membrane fatty acid composition of *H. congolense* WG10. First, our findings seem to align with the homeoviscous principle, which argues that membrane lipidome remodeling is driven by the need to maintain fluidity within a narrow range (Sinensky, 1974; Ernst et al., 2016). We quantitatively estimated degree of unsaturation as double bond index (DBI). Higher DBI connotes higher unsaturation and by implication lower viscosity/higher fluidity (Berezhnoy et al., 2022). Due to kinks in their hydrocarbon chains caused by the presence of double bonds, unsaturated fatty acids pack at relatively low densities, hence, promote membrane transition to the disordered liquid-crystalline phase. As shown in Figure 3, *H. congolense* WG10 adapted to low and high salt stress by further desaturating its membrane lipidome thereby increasing the fluidity of the bilayer matrix. This was also the response when the cells were challenged by an abnormally high hydraulic retention time (HRT) when growing at optimal temperature and salinity (Figure 3B). Fluidity determines ease of lateral diffusion of macromolecules in the bilayer matrix (Ballweg et al., 2020), and hence affects the spatial orientation, folding and functions of membrane proteins. Perhaps, *H. congolense* WG10 increases bilayer fluidity to spatially reorient thus functionalize or inhibit a cohort of membrane proteins such as sensors, kinases, channels, and transporters. Moreover, under high salinity stress, an increase in membrane fluidity might be necessary to counteract the gelation effect of monovalent cations (Russell, 1989; Seddon, 1990; Imhoff and Thiemann, 1991). However, the homeoviscous principle does not explain why *H. congolense* WG10 opted to increase membrane fluidity with polyunsaturated fatty acids (PUFAs) and not monounsaturated fatty acids. In fact, monounsaturation is sufficient for a bacterium to achieve its desired level of bilayer fluidity as introducing more than one double bond into a membrane fatty acid moiety exerts no additive effect on liquid-crystalline to gel transition (Russel and Nichols, 1999).

This gap in logic can be filled by the second hypothesis of membrane lipidome remodeling – the homeophasic principle. This principle holds that lipidome readjustment is geared toward controlling phase behavior (Linden et al., 1973). Membrane lipids can self-assemble into other supramolecular structures besides the bilayer, including micelles, cubic and hexagonal phases (Ernst et al., 2016). Predominance of non-bilayer phases negatively affects the biophysical properties and functions of the membrane (Russel and Nichols, 1999). While monounsaturated phospholipids favor the formation of non-bilayer phases, PUFAs allow just enough molecular motion to provide fluidity while preventing deleterious transition to inverted phases (Russel and Nichols, 1999). Therefore, it is apparent that *H. congolense* WG10 achieves sufficient fluidity while maintaining its bilayer structure under stress by increasing membrane PUFA composition.

Beyond regulating fluidity and phase behavior, PUFAs are known to alter other mechanical and biophysical properties of the membrane, including elasticity, thickness and curvature (Bruno et al., 2007). Membrane elasticity of *H. congolense* WG10, which we experimentally measured using atomic force microscopy (AFM), did not significantly vary with either HRT or salinity. On the other hand, we used mean phospholipid chain length as a quantitative estimate of bilayer thickness and found it to be significantly variant and positively correlated with membrane PUFA composition across gradients of salinity and hydraulic retention time (Figures 3–5). Hydrophobic thickness of bilayer membranes affects bending rigidity (Bermúdez et al., 2004), permeability (Discher et al., 1999) and elasticity (Bermudez et al., 2002). These properties, in turn, modulate the configuration and functions of membrane proteins including transporters and channels (Bruno et al., 2007). Hence, through several possible mechanistic and chemical processes, polyunsaturated fatty acids stabilize the bilayer membrane of *H. congolense* WG10 and endow it with biophysical attributes needed for adaptation to salinity-and HRT-induced perturbations. Differential gene expression analysis, proteomics and/or lipidomics investigations would shed more light on these underlying mechanisms.

## Conclusion

For *H. congolense* WG10 which persists in hydraulically fractured shale wells, salinity and hydraulic retention time (HRT) significantly influence membrane fatty acid composition and mechanics, and therefore, alter bilayer biophysics. Under non-optimal salinities, *H. congolense* WG10 increases the fluidity and thickness of the plasma membrane by elevating its PUFA composition. On the other hand, the effects of HRT on membrane fatty acid chemistry in *H. congolense* is less pronounced and discriminated by salinity level. The functions of the membrane, which include transport, metabolism, respiration, and cell-surface interactions, rely on the maintenance of optimal biophysical states. This study has demonstrated, under a simulated laboratory setting, how salinity and well flow rates affect the plasma membrane fatty acid chemistry of persistent shale taxa. This fundamental mechanistic insight will underlie efforts toward advancing our understanding of how environmental and engineered factors influence the physical and biogeochemical equilibria of subsurface hydrocarbon systems by inducing physiologically relevant changes in membrane features of resident taxa.

## Data availability statement

The original contributions presented in the study are included in the article/Supplementary material, further inquiries can be directed to the corresponding author.

## Author contributions

PM conceived and designed the study. JA, FC, and CU conducted the laboratory experiments. CU and FC processed the raw MS data. AB and YS conducted the AFM studies and processed the data. CU performed the statistical analyses and wrote the first draft of the manuscript. CU and PM developed the manuscript figures. All authors revised, read, and approved the submitted version of the manuscript.

## Funding

This work was funded by the U.S. Department of Energy, Office of Science, Office of Biological and Environmental Research (BER), and the Established Program to Stimulate Competitive Research (EPSCoR) (award number DESC0019444).

## Conflict of interest

FC is currently employed by New England Biolabs, Ipswich, MA, United States. JA is employed by Sanborn, Head and Associates Inc., Concord, NH, United States.

The remaining authors declare that the research was conducted in the absence of any commercial or financial relationships that could be construed as a potential conflict of interest.

## Publisher’s note

All claims expressed in this article are solely those of the authors and do not necessarily represent those of their affiliated organizations, or those of the publisher, the editors and the reviewers. Any product that may be evaluated in this article, or claim that may be made by its manufacturer, is not guaranteed or endorsed by the publisher.
