# Supplementary material for: Salinity and hydraulic retention time induce membrane phospholipid acyl chain remodeling in Halanaerobium congolense WG10 and mixed cultures from hydraulically fractured shale wells
Source: Front Microbiol. 2022 Nov 10;13:1023575. doi: 10.3389/fmicb.2022.1023575 (PMC9687094; doi:10.3389/fmicb.2022.1023575)
Supplement: Supplementary file 2 [file Data_Sheet_2.PDF]

Supplementary Material

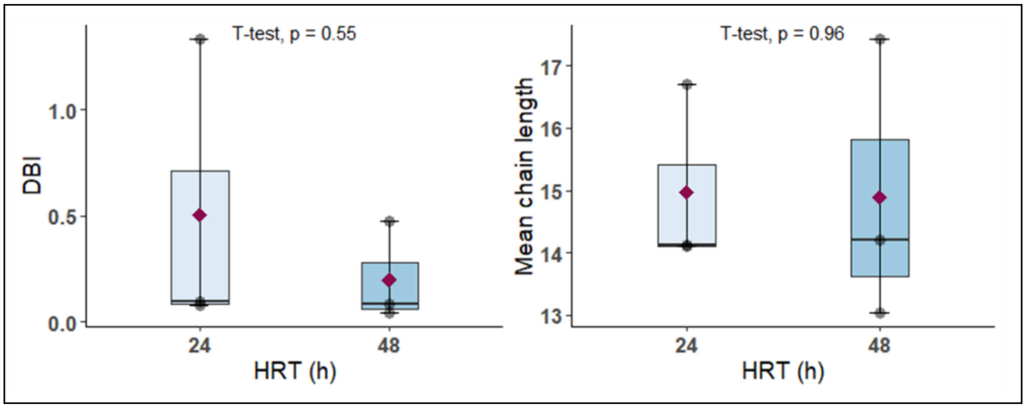

Figure S1. Mean chain length and double bond index (DBI) of membrane phospholipids in the mixed cultures of persistent shale taxa did not vary significantly with hydraulic retention time (HRT).

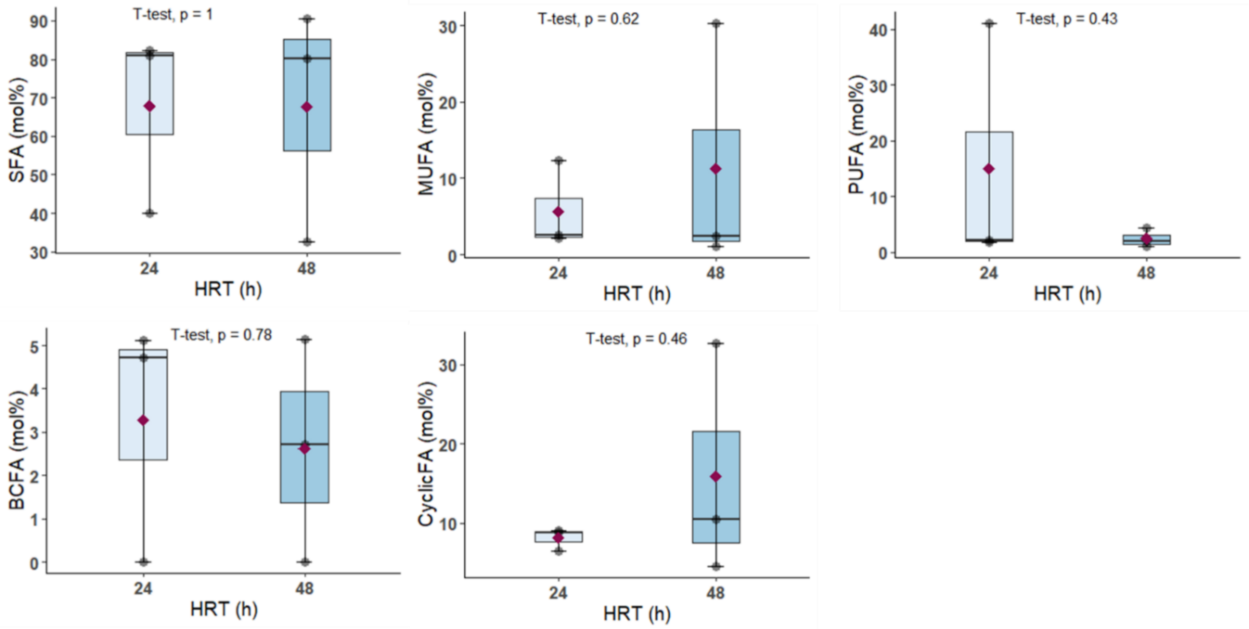

Figure S2. Membrane phospholipid fatty acid classes in the mixed cultures of persistent shale taxa did not vary significantly with hydraulic retention time (HRT).
